# RMTLysPTM: recognizing multiple types of lysine PTM sites by deep analysis on sequences

**DOI:** 10.1093/bib/bbad450

**Published:** 2023-12-08

**Authors:** Lei Chen, Yuwei Chen

**Affiliations:** College of Information Engineering, Shanghai Maritime University, Shanghai 201306, People’s Republic of China; College of Information Engineering, Shanghai Maritime University, Shanghai 201306, People’s Republic of China

**Keywords:** post-translation modification, acetylation, crotonylation, methylation, succinylation, multi-label classification

## Abstract

Post-translational modification (PTM) occurs after a protein is translated from ribonucleic acid. It is an important living creature life phenomenon because it is implicated in almost all cellular processes. Identification of PTM sites from a given protein sequence is a hot topic in bioinformatics. Lots of computational methods have been proposed, and they provide good performance. However, most previous methods can only tackle one PTM type. Few methods consider multiple PTM types. In this study, a multi-label classification model, named RMTLysPTM, was developed to recognize four types of lysine (K) PTM sites, including acetylation, crotonylation, methylation and succinylation. The surrounding sites of a lysine site were selected to constitute a peptide segment, representing the lysine at the center. Deep analysis was conducted to count the distribution of 2-residues with fixed location across the four types of lysine PTM sites. By aggregating the distribution information of 2-residues in one peptide segment, the peptide segment was encoded by informative features. Furthermore, a prediction engine that can precisely capture the traits of the above representations was designed to recognize the types of lysine PTM sites. The cross-validation results on two datasets (Qiu and CPLM training datasets) suggested that the model had extremely high performance and RMTLysPTM had strong generalization ability by testing it on protein Q16778 and CPLM testing datasets. The model was found to be generally superior to all previous models and those using popular methods and features. A web server was set up for RMTLysPTM, and it can be accessed at http://119.3.127.138/.

## INTRODUCTION

Post-translational modification (PTM) is a one of the largest stages in protein biosynthesis [1]. It occurs after a protein is translated from ribonucleic acid. PTM can change the physical and chemical properties of proteins through specific modifications, and it is implicated in almost all cellular processes. To date, hundreds of PTM types have been discovered and several of them have been identified to be related to diseases, such as cancer and neurological disorders [2]. Due to its importance in basic research and drug development, PTM is always a hot topic in protein science.

Among the discovered PTM types, the modification at lysine (K), also named K-PTM, is one of the most frequently observed and special PTM types. Lysine can be annotated by multiple types of PTM, including acetylation, biotinylation, butyrylation, crotonylation, methylation, propionylation, succinylation, ubiquitination and ubiquitin-like modifications [3]. In the past, biological experimental methods, such as mass spectroscopy and phosphor-specific antibody, were used to determine the PTM types. A solid determination can be obtained through these methods. However, their defect is very evident. Much time and high cost are needed to conduct these methods. With the coming of post-genome era, a great deal of protein sequences have emerged. These methods cannot process many sequences in time. Thus, designing fast and reliable methods to deal with such problem is needed.

In the past 20 years, *in silico* methods have become an alternative method to recognize PTM types. Several computational methods have been proposed to identify different lysine modifications, including acetylation [4–8], crotonylation [9–13], methylation [14–17] and succinylation [18–22]. These methods investigate one type of lysine PTM sites individually. For example, studies on acetylation did not consider crotonylation nor methylation. Combining the identification of different types of lysine PTM sites into a unified method is feasible because these types of modification involve lysine. In 2016, Qiu *et al*. [3] proposed the first computational method, iPTM-mLys, to identify four types of lysine PTM sites, including acetylation, crotonylation, methylation and succinylation. This method contains four procedures. Each procedure is in charge of identifying one type of lysine PTM site. A simple undersampling scheme is used to tackle the imbalanced problem. The results of the four procedures are combined as the final output of iPTM-mLys. Later, the following three methods were designed in a similar manner: predML-Site [23], mLysPTMpred [24] and iMul-kSite [25]; they improved iPTM-mLys by employing more powerful sampling schemes and more suitable single-label classification algorithms. The above methods have a common trait. They tackle four types of lysine PTM individually and divide the problem into four binary classification problems. Thus, they ignore the mutual influence of different types of lysine PTM sites. The other two methods, CNN + SGT [26] and MLysPRED [27], are designed in different manners. They extract more features from the sequences and directly apply multi-label classification algorithms [CNN in CNN + SGT and multi-label K-nearest neighbor classification algorithm (MLKNN) in MLysPRED] to make prediction. These two methods employ the sampling schemes to deal with imbalanced problems. The performance of all the existing methods was tested on the dataset reported in Qiu *et al*.’s study. However, it was not very high. The absolute true did not exceed 0.9, thus they could be improved.

In this study, data on lysine PTM sites collected in Qiu *et al*.’s study [3] were adopted, and they are denoted as Qiu dataset. By applying the sliding window technique, a peptide segment with 27 sites was obtained for each lysine. The distribution information of 2-residues with fixed location across four types of lysine PTM sites was counted by deep analysis on the sequences of peptide segments, and this information was further used to encode each peptide segment. On the basis of such representation, a prediction engine that can capture the traits of the representation was specially designed to recognize the types of lysine PTM sites. The developed model was called RMTLysPTM. Cross-validation tests indicated that the model had extremely high performance and strong generalization ability because it provided competitive prediction results on protein Q16778. The comparison results suggested that RMTLysPTM generally outperformed all the existing previous models and other models built using popular methods and features. Furthermore, RMTLysPTM was tested on two recently proposed datasets, CPLM training and testing datasets, and the outcomes indicated the high performance of RMTLysPTM.

## MATERIALS AND METHODS

**Figure 1:**
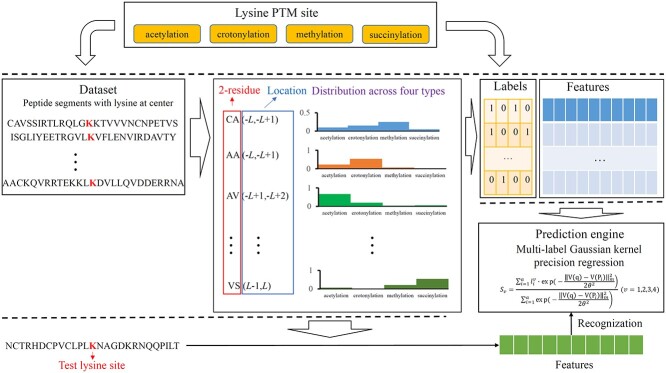
Flow chart of the RMTLysPTM. Four types of lysine PTM sites are considered, including acetylation, crotonylation, methylation and succinylation. The peptide segments containing lysine at center are used to represent the lysine. From the given dataset, the distribution information of 2-residue with fixed location is counted across four types and this information is adopted to encode peptide segments. A novel prediction engine is designed to recognize the types of lysine PTM sites.

The entire procedures for developing RMTLysPTM are illustrated in Figure 1. The detailed descriptions on each procedure are listed in this section.

### Benchmark dataset

**Figure 2:**
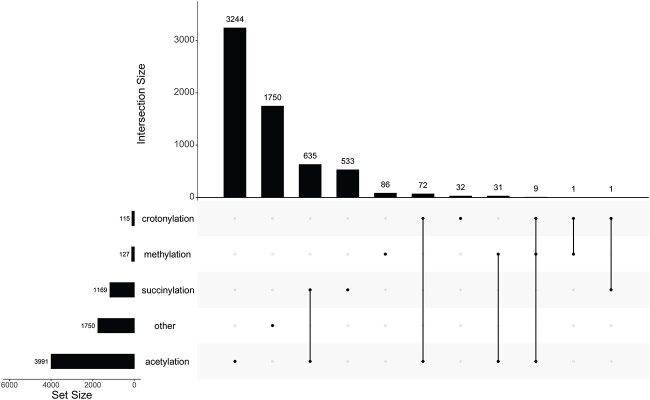
Upset graph to illustrate four types of lysine PTM sites in Qiu dataset. Several lysine sites belong to two types, only nine sites belong to three types and no sites belong to all four types. 1750 sites do not belong to any types.

The lysine PTM sites investigated in this study were retrieved from the study of Qiu *et al*. [3], who collected 6394 sites from UniProt (http://www.uniprot.org/). These sites comprise the dataset, denoted as *S*, and they involved 1763 human proteins. In accordance with the original study, these lysine PTM sites were divided into four PTM types: acetylation, crotonylation, methylation and succinylation. However, several sites were not annotated by these PTM types. These sites constitute the fifth class, termed as ‘other’. An upset graph was plotted to show the intersection of sites annotated by any of the four types or no type, as shown in Figure 2. The results showed that acetylation sites were annotated the most, followed by succinylation sites, whereas crotonylation and methylation sites were much less annotated than the above two types. Several sites belong to two or three types of PTM sites, whereas no sites belong to all four types. Clearly, recognizing the types of lysine PTM sites is a multi-label classification problem. In addition, 1750 lysine sites do not belong to any of the above PTM types. In the following formulation, the sets consisting of acetylation, crotonylation, methylation and succinylation sites were denoted as $S\left(\mathrm{acetylation}\right)$, $S\left(\mathrm{crotonylation}\right)$, $S\left(\mathrm{methylation}\right)$ and $S\left(\mathrm{succinylation}\right)$, respectively, whereas the other sites constitute the set $S\left(\mathrm{other}\right)$. Accordingly, the dataset *S* can be formulated by


(1) \begin{align*} S=S\left(\mathrm{acetylation}\right)\cup S\left(\mathrm{crotonylation}\right)\cup S\left(\mathrm{methylation}\right)\cup\nonumber \\S\left(\mathrm{succinylation}\right)\cup S\left(\mathrm{other}\right). \end{align*}

The lysine site only refers to a single amino-acid residue lysine in a protein sequence. Evidently, this information is insufficient to construct classification models. The sliding window technique is widely used in the field of PTM, and it was also employed in this study. From a protein sequence, the peptide segments that contain lysine at the center are extracted as follows:


(2) \begin{equation*} P\left(\mathrm{K}\right)={R}_{-L}{R}_{-L+1}\cdots{R}_{-1}K{R}_1\cdots{R}_{L-1}{R}_L, \end{equation*}

where *L* refers to the sliding window size, which was set to 13 in this study, as suggested in Qiu *et al*.’s study [3]. Such peptide segment clearly contains three parts: (i) lysine, (ii) upstream *L* residues of lysine in the sequence and (iii) downstream *L* residues of lysine in the sequence. In particular, if the upstream or downstream residues are less than *L*, the nearest residue is adopted to fill the locations. Finally, the dataset *S* consisting of 6394 peptide segments with length 27 was obtained, each of which contained lysine at the center. These peptide segments can be found in the supplementary files of Qiu *et al*.’s study [3].

**Figure 3:**
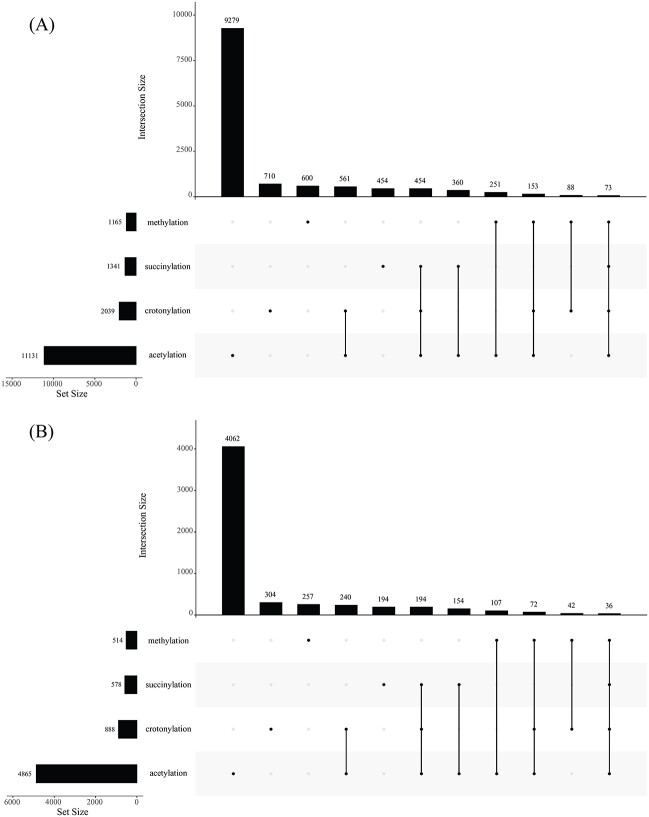
Upset graph to illustrate four types of lysine PTM sites in CPLM training and testing datasets. (**A**) Upset graph for CPLM training dataset. (**B**) Upset graph for CPLM testing dataset.

A recently constructed dataset used in [27], which was retrieved from CPLM 4.0 [28], a data resource for various PTMs specifically occurring at the side-chain amino group of lysine residues in proteins, was further employed to fully test the proposed model. A total of 18 978 human protein sequences containing at least one lysine modified by acetylation, crotonylation, methylation or succinylation were downloaded from CPLM 4.0. After the data cleaning procedure described in [27] was conducted, 7057 human protein sequences were kept. The lysine sites modified by acetylation, crotonylation, methylation or succinylation were divided into two parts, 70% of them constituted the training dataset, whereas the remaining 30% data comprised the testing dataset. An upset graph for the lysine sites in the training and test datasets was separately plotted, as shown in Figure 3. Such dataset was much larger than the previous dataset, indicating that the test results on such dataset were more reliable. Furthermore, several lysine sites can be modified by all four types, and the lysine sites that cannot be modified by any PTM type were not included, which are the major differences from the previous dataset. Testing these two different datasets can fully evaluate the model’s performance. For easy description, the training and testing datasets reported in [27] are called CPLM training and testing datasets, respectively, whereas the dataset obtained from Qiu *et al*.’s study [3] is called Qiu dataset. The CPLM training and testing datasets can be obtained at http://47.100.136.41:8181/dataSet.

### Feature construction

Feature representation is an important step to develop efficient classification models. In accordance with the Benchmark dataset section, the upstream and downstream residues of the investigated lysine sites in Qiu dataset were selected to constitute a peptide segment with length 27. How to extract informative features from such peptide segment was a challenging problem. Inspired by *k*-mer in DNA research, the peptide segment was separated into several *k*-residues consisting of continuous *k*-residues in the peptide segment. For example, given a peptide segment ‘CAVSSIRTLRQLGKKTVVVNCNPETVS’, its *k*-residues were ‘CA, AV, VS, …, VS’ when *k* = 2. In this study, a novel scheme was proposed to extract informative features based on the *k*-residues. A huge number of combinations would exist when *k* is large because 20 different amino acids are present. Accordingly, *k* was set to 2 in this study.

${\mathrm{S}}_{tr}=\left\{{P}_1,{P}_2,\cdots, {P}_a\right\}$ is assumed to be a training dataset, where *a* is the number of training peptide segments and ${P}_i$ is the *i*-th peptide segment containing lysine at the center. These training peptide segments were classified into five subsets in accordance with the type of lysine PTM site. The first four subsets contain the peptide segments of acetylation, crotonylation, methylation and succinylation, denoted as ${\mathrm{S}}_{tr}\left(\mathrm{acetylation}\right)$, ${\mathrm{S}}_{tr}\left(\mathrm{crotonylation}\right)$, ${\mathrm{S}}_{tr}\left(\mathrm{methylation}\right)$ and ${\mathrm{S}}_{tr}\left(\mathrm{succinylation}\right)$, respectively, and the last subset contained the remaining peptide segments, indicated by ${\mathrm{S}}_{tr}\left(\mathrm{other}\right)$. For any 2-residue, such as ‘CA’, if it frequently occurs in the peptide segments of one type of PTM site, the test peptide segment containing such 2-residue is more likely annotated by such PTM type. However, the location of the 2-residue is an important information, which should be also included. In detail, the same 2-residue close to the center lysine and far away from the center lysine should be considered as different 2-residues. Thus, the 2-residue was denoted as $\alpha \beta \left(i,i+1\right)$, where $\alpha$ and $\beta$ stand for two amino acids in 2-residue, and $i\in \left\{-L,-L+1,\cdots, -1,0,1,\cdots, L-1\right\}$ represent the locations of $\alpha$ and $\beta$ in the peptide segment, as the subscripts in Eq. 2. For $\alpha \beta \left(i,i+1\right)$, the number of training peptide segments, which contain it at the same location, is counted. Such entry is represented by $N\left(\alpha \beta \left(i,i+1\right)\right)$, i.e.


(3) \begin{equation*} N\left(\alpha \beta \left(i,i+1\right)\right)={\sum}_{k=1}^a\Delta \left(\alpha \beta \left(i,i+1\right),{P}_k\right), \end{equation*}

where $\Delta \left(\alpha \beta \left(i,i+1\right),{P}_k\right)$ is defined as


(4) \begin{align*}& \Delta \left(\alpha \beta \left(i,i+1\right),{P}_k\right)\nonumber\\&=\left\{\begin{array}{ll}1& {P}_k\ \mathrm{contains}\ \alpha \beta \left(i,i+1\right)\ \mathrm{at}\ \mathrm{the}\ \mathrm{same}\ \mathrm{location}\\{}0& \mathrm{otherwise}\end{array}\right. \end{align*}

Furthermore, the number of peptide segments in four subsets, i.e. ${S}_{tr}\left(\mathrm{acetylation}\right)$, ${S}_{tr}\left(\mathrm{crotonylation}\right)$, ${S}_{tr}\left(\mathrm{methylation}\right)$ and ${S}_{tr}\left(\mathrm{succinylation}\right)$, is counted as follows:


(5) \begin{equation*} \left\{\begin{array}{l}{N}_{\mathrm{acetylation}}\left(\alpha \beta \left(i,i+1\right)\right)={\sum}_{P\in{\mathrm{S}}_{tr}\left(\mathrm{acetylation}\right)}\Delta \left(\alpha \beta \left(i,i+1\right),P\right)\\{}{N}_{\mathrm{crotonylation}}\left(\alpha \beta \left(i,i+1\right)\right)={\sum}_{P\in{\mathrm{S}}_{tr}\left(\mathrm{crotonylation}\right)}\Delta \left(\alpha \beta \left(i,i+1\right),P\right)\\{}{N}_{\mathrm{methylation}}\left(\alpha \beta \left(i,i+1\right)\right)={\sum}_{P\in{\mathrm{S}}_{tr}\left(\mathrm{methylation}\right)}\Delta \left(\alpha \beta \left(i,i+1\right),P\right)\\{}{N}_{\mathrm{succinylation}}\left(\alpha \beta \left(i,i+1\right)\right)={\sum}_{P\in{\mathrm{S}}_{tr}\left(\mathrm{succinylation}\right)}\Delta \left(\alpha \beta \left(i,i+1\right),P\right)\end{array}\right. \end{equation*}

The above entries reflect the distribution of $\alpha \beta \left(i,i+1\right)$ across four types of PTM sites. If one of the above entry is large, the test peptide segment containing $\alpha \beta \left(i,i+1\right)$ at the same location is more likely to be annotated by the corresponding PTM type. Meanwhile, if the above four entries are small, the test peptide segment may not be annotated by any PTM type. Thus, these entries are useful information to recognize the types of lysine PTM sites. However, direct use of them is not a perfect method because the ranges are relatively different for various 2-residues. Thus, they were further refined as follows:


(6) \begin{equation*} \left\{\begin{array}{l}{\rho}_{\mathrm{acetylation}}\left(\alpha \beta \left(i,i+1\right)\right)={N}_{\mathrm{acetylation}}\left(\alpha \beta \left(i,i+1\right)\right)/N\left(\alpha \beta \left(i,i+1\right)\right)\\{}{\rho}_{\mathrm{crotonylation}}\left(\alpha \beta \left(i,i+1\right)\right)={N}_{\mathrm{crotonylation}}\left(\alpha \beta \left(i,i+1\right)\right)/N\left(\alpha \beta \left(i,i+1\right)\right)\\{}{\rho}_{\mathrm{methylation}}\left(\alpha \beta \left(i,i+1\right)\right)={N}_{\mathrm{methylation}}\left(\alpha \beta \left(i,i+1\right)\right)/N\left(\alpha \beta \left(i,i+1\right)\right)\\{}{\rho}_{\mathrm{succinylation}}\left(\mathrm{\alpha} \mathrm{\beta} \left(i,i+1\right)\right)={N}_{\mathrm{succinylation}}\left(\alpha \beta \left(i,i+1\right)\right)/N\left(\alpha \beta \left(i,i+1\right)\right)\end{array}\right. \end{equation*}

Entries in Eq. 6 indicate the proportions of peptide segments that contain $\alpha \beta \left(i,i+1\right)$ at the same location, which are all between 0 and 1. These values are suitable to represent $\alpha \beta \left(i,i+1\right)$. Accordingly, $\alpha \beta \left(i,i+1\right)$ can be represented by a four-dimension vector, formulated as


(7) \begin{align*} \mathrm{V}\left(\alpha \beta \left(i,i+1\right)\right)=[{\rho}_{\mathrm{acetylation}}\left(\alpha \beta \left(i,i+1\right)\right),\kern0.5em {\rho}_{\mathrm{crotonylation}}\left(\alpha \beta \left(i,i+1\right)\right),\nonumber\\{\rho}_{\mathrm{methylation}}\left(\alpha \beta \left(i,i+1\right)\right),{\rho}_{\mathrm{succinylation}}\left(\alpha \beta \left(i,i+1\right)\right)]^T \end{align*}

For a 2-residue KA(−*L*, −*L* + 1), the number of peptide segments with ${R}_{-L}=K$ and ${R}_{-L+1}=A$ (Eq. 2 shows the formulation of peptide segment) in the training dataset is counted. When this value is 500, that is, $N\left( KA\left(-L,-L+1\right)\right)=500$, then the numbers of such peptide segments containing lysine sites modified by acetylation, crotonylation, methylation and succinylation are further counted. When they are 50, 10, 400 and 0, i.e. ${N}_{\mathrm{acetylation}}\left( KA\left(-L,-L+1\right)\right)=50$, ${N}_{\mathrm{crotonylation}}\left( KA\left(-L,-L+1\right)\right)=10$, ${N}_{\mathrm{methylation}}\left( KA\left(-L,-L+1\right)\right)=400$ and ${N}_{\mathrm{succinylation}}\left( KA\left(-L,-L+1\right)\right)=0$, respectively, the above values induce four items: ${\rho}_{\mathrm{acetylation}}\left( KA\left(-L,-L+1\right)\right)=\frac{50}{500}=0.1$, ${\rho}_{\mathrm{crotonylation}}\left( KA\left(-L,-L+1\right)\right)=\frac{10}{500}=0.02$, ${\rho}_{\mathrm{methylation}}( KA(-L,-L+1))=\frac{400}{500}=0.8$ and ${\rho}_{\mathrm{succinylation}}\left( KA\left(-L,-L+1\right)\right)=\frac{0}{500}=0$. These items constitute a four-dimension vector ${\left[0.1,\mathrm{0.02,0.8,0}\right]}^T$ to represent *KA*(−*L*, −*L* + 1). For a training or test peptide segment with ${R}_{-L}=K$ and ${R}_{-L+1}=A$, this vector is used to represent these two residues in the peptide segment. By deep analysis of the training peptide segments, all 2-residues at each location can be encoded into a four-dimension vector. These features contain not only the location information of the 2-residue in training peptide segments but also the label information of training peptide segments. They are useful to determine the types of lysine PTM sites for a given test lysine alone with its peptide segment.

For a test or training peptide segment of length *L*, all its 2-residues were selected except those containing the center lysine, $2\times \left(L-1\right)$ 2-residues in total. As mentioned above, each 2-residue can be represented by a four-dimension vector. The vectors of all collected 2-residues are aggregated together to generate the representation of the peptide segment. For the peptide segment listed in Eq. 2, its feature vector is formulated as follows:


(8) \begin{align*} \mathrm{V}\left(\mathrm{P}\right)=\mathrm{V}\left({R}_{-L}{R}_{-L+1}\left(-L,-L+1\right)\right)\oplus \mathrm{V}\nonumber\\\left({R}_{-L+1}{R}_{-L+2}\left(-L+1,-L+2\right)\right)\oplus \cdots \oplus \mathrm{V}\left({R}_{L-1}{R}_L\left(L-1,L\right)\right), \end{align*}

where $\oplus$ is the concatenation operation. Accordingly, each peptide segment was represented by $8\times \left(L-1\right)$ features. Given that the peptide segments in Qiu dataset were obtained by setting *L* = 13, they were represented by 96 features. For easy description, these features were called distribution features.

### Prediction engine

Selecting or designing a proper classification algorithm is important when building efficient classification models. A novel algorithm that can capture the traits of such representation was designed on the basis of the feature representation of the peptide segment containing lysine at the center. As mentioned in Benchmark dataset section, this algorithm can process samples with multiple labels.

Multi-label Gaussian kernel regression (ML-GKR) is a widely used algorithm to set up multi-label classification models [29–34]. In the present study, its variation was designed so that the new designed algorithm can more efficiently process above-constructed features. In accordance with the feature vector listed in Eq. 8, it aggregated four features of 2-residues in the sequence in order. The first feature was for acetylation, followed by those for crotonylation, methylation and succinylation, respectively. Thus, each component in the final feature vector is highly related to a certain type of PTM sites (acetylation, crotonylation, methylation or succinylation, which were termed as labels when constructing classification models). In ML-GKR, the score for each label is calculated using all features because these features are not special for any labels. In view of the trait of the feature vector, the score for one label can be calculated on the basis of the features related to this label only, thereby improving the accuracy of the score. The new method is referred to as multi-label Gaussian kernel precision regression (ML-GKPR).

Each peptide segment in a given training dataset containing *a* peptide segments, denoted by ${S}_{tr}=\left\{{P}_1,{P}_2,\cdots, {P}_a\right\}$, can be represented by an $8\times \left(L-1\right)$-dimension feature vector according to Eq. 8. The feature vector of the *i*-th peptide segment is formulated as follows:


(9) \begin{equation*} \mathrm{V}\left({\mathrm{P}}_i\right)={\left[{F}_i^1,{F}_i^2,\cdots, {F}_i^{8\left(L-1\right)}\right]}^T, \end{equation*}

and its observed labels can be formulated by a four-dimension binary vector, denoted by


(10) \begin{equation*} {L}_i={\left[{l}_i^1,{l}_i^2,{l}_i^3,{l}_i^4\right]}^T, \end{equation*}

where ${l}_i^j\ \left(1\le j\le 4\right)$ is defined as follows:


(11) \begin{equation*} {l}_i^1=\left\{\begin{array}{ll}+1& \mathrm{if}\ {\mathrm{P}}_i\ \mathrm{is}\ \mathrm{annotated}\ \mathrm{by}\ \mathrm{acetylation}\\{}-1& \mathrm{otherwise}\end{array}\right. \end{equation*}

(12) \begin{equation*} {l}_i^2=\left\{\begin{array}{ll}+1& \mathrm{if}\ {\mathrm{P}}_i\ \mathrm{is}\ \mathrm{annotated}\ \mathrm{by}\ \mathrm{crotonylation}\\{}-1& \mathrm{otherwise}\end{array}\right. \end{equation*}

(13) \begin{equation*} {l}_i^3=\left\{\begin{array}{ll}+1& \mathrm{if}\ {\mathrm{P}}_i\ \mathrm{is}\ \mathrm{annotated}\ \mathrm{by}\ \mathrm{methylation}\\{}-1& \mathrm{otherwise}\end{array}\right. \end{equation*}

(14) \begin{equation*} {l}_i^4=\left\{\begin{array}{ll}+1& \mathrm{if}\ {\mathrm{P}}_i\ \mathrm{is}\ \mathrm{annotated}\ \mathrm{by}\ \mathrm{succinylation}\\{}-1& \mathrm{otherwise}\end{array}\right. \end{equation*}

For a query peptide segment *q* containing lysine at the center, its feature vector is formulated by


(15) \begin{equation*} \mathrm{V}\left(\mathrm{q}\right)={\left[{F}_q^1,{F}_q^2,\cdots, {F}_q^{8\left(L-1\right)}\right]}^T. \end{equation*}

Its label vector can be determined as follows:


(16) \begin{equation*} {L}_q={\left[{l}_q^1,{l}_q^2,{l}_q^3,{l}_q^4\right]}^T. \end{equation*}

The score for each label is calculated as follows:


(17) \begin{equation*} {S}_v=\frac{\sum_{i=1}^a{l}_i^v\bullet \exp \left(-\frac{{\left\Vert \mathrm{V}\left(\mathrm{q}\right)-\mathrm{V}\left({\mathrm{P}}_i\right)\right\Vert}_m^2}{2{\theta}^2}\right)}{\sum_{i=1}^a\exp \left(-\frac{{\left\Vert \mathrm{V}\left(\mathrm{q}\right)-\mathrm{V}\left({\mathrm{P}}_i\right)\right\Vert}_m^2}{2{\theta}^2}\right)},\kern2.5em v=1,2,3,4 \end{equation*}

where $\theta$ is a parameter, and ${\left\Vert \mathrm{V}\left(\mathrm{q}\right)-\mathrm{V}\left({\mathrm{P}}_i\right)\right\Vert}_m^2$ is defined as


(18) \begin{equation*} {\left\Vert \mathrm{V}\left(\mathrm{q}\right)-\mathrm{V}\left({\mathrm{P}}_i\right)\right\Vert}_m^2={\sum}_{j=0}^{2L-3}{\left({F}_q^{4j+v}-{F}_i^{4j+v}\right)}^2. \end{equation*}

According to ${S}_v$, ${l}_q^v$ is determined in the following manner:


(19) \begin{equation*} {l}_q^v=\left\{\begin{array}{ll}+1& \mathrm{if}\ {S}_v\ge 0\\{}-1& \mathrm{otherwise}\end{array}\right.. \end{equation*}

The classification model developed using the above features and prediction engine is called RMTLysPTM in this study.

### Performance evaluation

Several validation methods can be used to evaluate the performance of classification models. Cross-validation is one of the most widely used methods. In this method, samples are equally and randomly divided into *K* parts. Each part is selected as a test set one by one and the remaining parts comprise the training set. The model built on the training set is applied to the test set. The average performance on each part is selected as the final performance of the classification model. In general, *K* is set to 5 or 10. Here, 5 was selected, i.e. 5-fold cross-validation was adopted to assess the performance of all classification models. According to the feature construction scheme mentioned in Feature construction section, the procedures are highly related to the training samples, that is, the representation of the same sample is not the same in different rounds of cross-validation. Therefore, samples were divided into five parts, and then each sample was encoded on the basis of this division. In this manner, the information of test samples could be rigorously excluded when training the classification model.

For a multi-label classification model, its cross-validation results can be counted as several measurements. Here, the same measurements used in previous studies [3, 23–27] were selected for easy comparison. These measurements included aiming, coverage, accuracy, absolute true and absolute false, which all have wide applications in evaluating the performance of multi-label classification models [35–37]. Some notations are necessary to show how to compute them. For a dataset containing *N* samples and *M* labels, the observed labels of the *i*-th sample constitute the set ${L}_i$, whereas its predicted labels comprise the set ${L}_i^{\ast }$. Then, the above measurements can be formulated as follows:


(20) \begin{equation*} \left\{\begin{array}{l}\mathrm{Aiming}=\frac{1}{N}\sum_{i=1}^N\frac{\left\Vert{L}_i\cap{L}_i^{\ast}\right\Vert }{\left\Vert{L}_i^{\ast}\right\Vert}\\{}\mathrm{Coverage}=\frac{1}{N}\sum_{i=1}^N\frac{\left\Vert{L}_i\cap{L}_i^{\ast}\right\Vert }{\left\Vert{L}_i\right\Vert}\\{}\mathrm{Accuracy}=\frac{1}{N}\sum_{i=1}^N\frac{\left\Vert{L}_i\cap{L}_i^{\ast}\right\Vert }{\left\Vert{L}_i\cup{L}_i^{\ast}\right\Vert}\\{}\mathrm{Absolute}\ \mathrm{true}=\frac{1}{N}\sum_{i=1}^N\Delta \left({L}_i,{L}_i^{\ast}\right)\\{}\mathrm{Absolute}\ \mathrm{false}=\frac{1}{N}\sum_{i=1}^N\frac{\left\Vert{L}_i\cup{L}_i^{\ast}\right\Vert -\left\Vert{L}_i\cap{L}_i^{\ast}\right\Vert }{M}\end{array}\right.\!\!\!\!\!\!, \end{equation*}

where $\Delta \left({L}_i,{L}_i^{\ast}\right)$ is an indicator, which is set to 1 if ${L}_i$ and ${L}_i^{\ast }$ are identical; otherwise, it is set to 0. Clearly, the higher the aiming, coverage, accuracy and absolute true, the higher the performance. On the contrary, i.e. a low absolute false value suggests high performance.

Besides the above overall measurements, some measurements that are defined on each label were employed. For one label, the samples having this label are termed as positive samples, whereas the others are considered as negative samples. Then, the sensitivity (SN), specificity (SP), accuracy (ACC), precision and F1-score for the *i*-th label can be calculated as follows:


(21) \begin{equation*} \left\{\begin{array}{l}\mathrm{SN}(i)=\frac{\mathrm{TP}(i)}{\mathrm{TP}(i)+\mathrm{FN}(i)}\\{}\mathrm{SP}(i)=\frac{\mathrm{TN}(i)}{\mathrm{TN}(i)+\mathrm{FP}(i)}\\{}\mathrm{ACC}(i)=\frac{\mathrm{TP}(i)+\mathrm{TN}(i)}{\mathrm{TP}(i)+\mathrm{FP}(i)+\mathrm{TN}(i)+\mathrm{FN}(i)}\\{}\mathrm{Precision}(i)=\frac{\mathrm{TP}(i)}{\mathrm{TP}(i)+\mathrm{FP}(i)}\\{}F1-\mathrm{score}(i)=\frac{2\times \mathrm{Recall}(i)\times \mathrm{Precision}(i)}{\mathrm{Recall}(i)+\mathrm{Precision}(i)}\end{array}\right.\!\!\!\!\!, \end{equation*}

where $\mathrm{TP}(i),\kern0.5em \mathrm{TN}(i),\kern0.5em \mathrm{FP}(i)$ and $\mathrm{FN}(i)$ stand for the true positive, true negative, false positive and false negative for the *i*-th label, respectively. Furthermore, ROC and PR curves are plotted to fully display the models’ performance on the *i*-th label, and the areas under these two curves are calculated, denoted by $\mathrm{AUROC}(i)$ and $\mathrm{AUPR}(i)$, respectively.

## RESULTS AND DISCUSSION

### Parameter optimization

**Figure 4:**
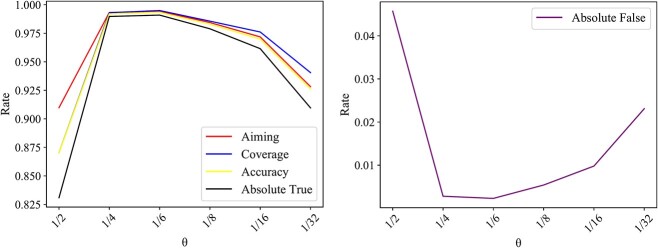
Performance of the multi-label classification models under different values of $\theta$ in the prediction engine on Qiu dataset. When $\theta =1/6$, the model provides the best performance on all five overall measurements.

For the prediction engine mentioned in Prediction engine section, $\theta$ is an important parameter, similar to that in ML-GBK. Several values (1/2, 1/4, 1/6, 1/8, 1/16 and 1/32) were attempted for this parameter, and the corresponding model was built, which was evaluated by 5-fold cross-validation. The overall performance of the models with different values of $\theta$ is listed in Figure 4. At first, with the decrease in $\theta$, the performance improved, and when $\theta =1/6$, the model yielded the best performance for all overall measurements. Then, the performance reduced with the decrease in $\theta$. Clearly, $1/6$ was the best setting for $\theta$. Thus, the model with $\theta =1/6$ was the proposed model of this study and called RMTLysPTM.

### Performance of RMTLysPTM

**Figure 5:**
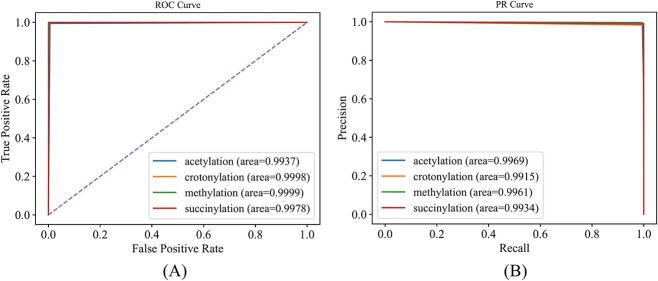
ROC and PR curves of RMTLysPTM for four types of lysine PTM sites in Qiu dataset. (**A**) ROC curves; (**B**) PR curves. The ROC and PR curves are nearly perfect and the AUROC and AUPR values are close to one, suggesting the extreme high performance of RMTLysPTM on four types of lysine PTM sites.

As mentioned in Parameter optimization section, $\theta$ was set to $1/6$ in RMTLysPTM. Its overall performance (measurements are listed in Eq. 20) under 5-fold cross-validation is listed in Table 1. Evidently, the performance was extremely high. In detail, aiming, coverage, accuracy and absolute true reached 0.9943, 0.9949, 0.9934 and 0.9909, respectively. All values were higher than 0.99. Meanwhile, the absolute false value was as low as 0.0023. The performance of the model on four types of PTM sites was evaluated. The measurements (cf. Eq. 21) are listed in Table 2. Similar to the overall performance, all measurements on the four types were very high. Most were higher than 0.99, and only two values were between 0.98 and 0.99. Evidently, the performance of RMTLysPTM on each type was very high. In addition, the ROC and PR curves on each type were plotted, as shown in Figure 5. All the AUROC and AUPR values were higher than 0.99, further proving the high performance of RMTLysPTM.

**Table 1:** Overall performance of RMTLysPTM on Qiu dataset

Measurement	RMTLysPTM (5-fold cross-validation)	RMTLysPTM excluding lysine sites without labels (5-fold cross-validation)	RMTLysPTM (strict 5-fold cross-validation)
Aiming	0.9943	0.9929	0.9306
Coverage	0.9949	0.9989	0.9604
Accuracy	0.9934	0.9925	0.9291
Absolute true	0.9909	0.9856	0.8955
Absolute false	0.0023	0.0036	0.0275

**Table 2:** Performance of RMTLysPTM on four types of lysine PTM sites in Qiu dataset

Type	Accuracy	Precision	Sensitivity	Specificity	F1-score
Acetylation	0.9937	0.9962	0.9947	0.9937	0.9950
Crotonylation	0.9997	0.9829	1.000	0.9997	0.9914
Methylation	0.9998	0.9922	1.000	0.9998	0.9961
Succinylation	0.9975	0.9881	0.9983	0.9989	0.9932

According to Figure 2, 1750 lysine PTM sites were not labeled by any type. Investigating whether these samples influence the performance of RMTLysPTM is necessary. Thus, these lysine sites were excluded, and RMTLysPTM was constructed again, which was called RMTLysPTM excluding lysine sites without labels to distinguish RMTLysPTM. Its performance under 5-fold cross-validation is listed in Table 1. The aiming, coverage, accuracy and absolute true values were 0.9929, 0.9989, 0.9925 and 0.9856, respectively, and the absolute false value was 0.0036. Compared with the performance of RMTLysPTM, which is listed in Table 1, such performance slightly reduced. Each measurement decreased or increased by less than 1%. These results suggested that the lysine sites without any PTM type provided limited influence for RMTLysPTM.

RMTLysPTM showed excellent performance for recognizing the types of lysine PTM sites regardless of whether the special lysine sites (without any PTM type) were included or not.

### Influence of sliding window size

In this study, the sliding window technique was adopted to generate the peptide segment for each lysine site. This technique is commonly used in PTM prediction. The sliding window size was set to 13 in RMTLysPTM (*L* = 13 in Eq. 2). Here, the sizes were set to 7, 9 and 11, and the performance of RMTLysPTM was evaluated with these sliding window sizes. The 5-fold cross-validation results are listed in Table 3. For easy comparison, the overall performance of RMTLysPTM with a size of 13 is also listed in this table. The aiming, coverage, accuracy and absolute true values followed an increasing trend with the increase in sliding window sizes, whereas the absolute false value followed a contrary trend. The findings implied that the performance of RMTLysPTM enhanced when the sliding window size increased. When the size reached 13, the performance of RMTLysPTM was sufficiently high that setting the size to larger than 13 was not necessary.

**Table 3:** Performance of RMTLysPTM under different sliding window sizes on Qiu dataset

Sliding window size (*L*)	Aiming	Coverage	Accuracy	Absolute true	Absolute false
7	0.9745	0.9742	0.9714	0.9653	0.0091
9	0.9866	0.9870	0.9850	0.9814	0.0048
11	0.9921	0.9929	0.9912	0.9888	0.0029
13	0.9943	0.9949	0.9934	0.9909	0.0023

### Strict test on RMTLysPTM

The general 5-fold cross-validation randomly and equally divided peptide segments into five parts. The peptide segments derived from the same protein sequence may be in the training and test datasets in a certain round of 5-fold cross-validation. In this case, the evaluation results may be overestimated. Thus, a strict 5-fold cross-validation was adopted to fully test RMTLysPTM.

**Figure 6:**
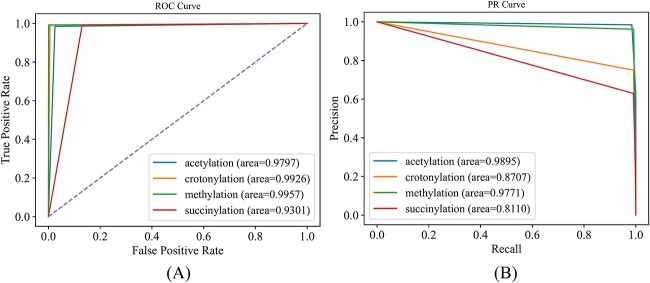
ROC and PR curves of RMTLysPTM for four types of lysine PTM sites in Qiu dataset under a strict test. (**A**) ROC curves; (**B**) PR curves. The AUROC and AUPR values are lower than those in Figure 5. The performance of RMTLysPTM on four types of lysine PTM sites reduced under a strict test. However, this performance is still relatively high.

All protein sequences were randomly and equally divided into five parts. In each round, the peptide segments derived from the sequences in one part were selected as test samples, whereas the others constituted the training samples. The peptide segments extracted from the same protein sequence can only be contained in the test dataset or training dataset, that is, the protein sequences involved in the test samples were not considered in the training of the model. This division was stricter than that in the general 5-fold cross-validation. RMTLysPTM was evaluated by such cross-validation method, and the results are listed in the last column of Table 1. The performance evidently reduced. The aiming, coverage, accuracy and absolute true values decreased to 0.9306, 0.9604, 0.9291 and 0.8955, respectively, whereas the absolute false value increased to 0.0275. Although such performance was inferior to that yielded by the general 5-fold cross-validation, it was still relatively high. The aiming, coverage, accuracy and absolute true values were still higher or close to/than 0.9. The performance of RMTLysPTM on the four PTM types under such test is provided in Table 4 and Figure 6. Most measurements in Table 4 were very high (≥0.9); three AUROC values were higher than 0.97, and two AUPR values were higher than 0.97, suggesting the high performance of RMTLysPTM. These results indicated that RMTLysPTM remained powerful in recognizing the types of lysine PTM sites from a new protein sequence.

**Table 4:** Performance of RMTLysPTM on four types of lysine PTM sites in Qiu dataset under a strict 5-fold cross-validation

Type	Accuracy	Precision	Sensitivity	Specificity	F1-score
Acetylation	0.9809	0.9847	0.9847	0.9847	0.9838
Crotonylation	0.9939	0.7500	0.9913	0.8539	0.8973
Methylation	0.9991	0.9618	0.9921	0.9767	0.9824
Succinylation	0.8918	0.6297	0.9906	0.7699	0.8205

### Extensive comparisons of other models

Some models were constructed using popular methods and features and compared with RMTLysPTM to indicate its superiority.

In RMTLysPTM, ML-GKR was modified and a new prediction engine, called ML-GKPR, which can precisely capture the traits of the representations of peptide segments, was constructed. Here, ML-GKPR was replaced with ML-GKR to set up another model. The parameter $\theta$ was tuned, and $\theta =1/4$ gave the best performance. Such model was evaluated by 5-fold cross-validation. The overall performance is listed in Table 5. The aiming, coverage, accuracy and absolute true values were 0.9806, 0.9790, 0.9775 and 0.9728, respectively, which were all lower than those of RMTLysPTM. The absolute false value was 0.0069, higher than that of RMTLysPTM. This model evidently provided lower performance than RMTLysPTM. Furthermore, the detailed performance of the model on the four PTM types is listed in Supplementary Table S1, whereas the ROC and PR curves are provided in Supplementary Figure S1A and B, respectively. Compared with the corresponding results of RMTLysPTM (Table 2 and Figure 5), RMTLysPTM provided better performance. All these results proved that ML-GKPR was more suitable to deal with the representation of peptide segments proposed in this study than ML-GKR.

**Table 5:** Performance of various models on Qiu dataset using popular methods and features

Feature	Prediction engine	Aiming	Coverage	Accuracy	Absolute true	Absolute false
Distribution feature	ML-GKPR	0.9943	0.9949	0.9934	0.9909	0.0023
Distribution feature	ML-GKR	0.9806	0.9790	0.9775	0.9728	0.0069
Distribution feature	RAKEL (Random forest)	0.9948	0.9946	0.9913	0.9784	0.0056
Distribution feature	RAKEL (Decision tree)	0.6772	0.6295	0.6295	0.5823	0.1199
PSSM feature	RAKEL (Random forest)	0.6114	0.5635	0.5624	0.5332	0.1406
PSSM feature	RAKEL (Decision tree)	0.5912	0.5365	0.5365	0.5167	0.1464

ML-GKR and ML-GKPR are algorithm adaption methods for building multi-label classification models. Another popular method is problem transformation, which transforms the original problem into multiple single-label classification problems. RAndom k-labELsets (RAKEL) [38] is one of the most classic problem transformation methods, and it was adopted in the present study to construct models. Random forest (RF) [39] and decision tree (DT) [40] were adopted as the base classification algorithm. RAKEL, RF and DT were implemented by Meka (http://waikato.github.io/meka/) [41]. The models using different base classification algorithms (RF or DT) were constructed and evaluated by 5-fold cross-validation. The evaluation results are listed in Table 5. When RF was adopted as the base classification algorithm, the model provided aiming, coverages, accuracy, absolute true and absolute false values of 0.9948, 0.9946, 0.9913, 0.9784 and 0.0056, respectively. The aiming value was slightly higher than that of RMTLysPTM, the coverage and accuracy values were slightly lower, the absolute true value was about 1.3% lower and the absolute false value was about 0.3% higher. In general, RMTLysPTM was more powerful than the RAKEL model. The performance of such RAKEL model on four PTM types (Supplementary Table S1 and Supplementary Figure S1C and D) indicated that it was generally inferior to RMTLysPTM. When DT was used as the base classification algorithm, the performance of such model was quite low. The aiming, coverage, accuracy and absolute true values were lower than 0.7, and the absolute false value was higher than 0.1. Evidently, the RAKEL model was much inferior to RMTLysPTM. The same conclusion can be found by observing the performance of the RAKEL model on the four PTM types (Supplementary Table S1 and Supplementary Figure S1E and F).

Besides ML-GKPR, the reason for the superiority of RMTLysPTM was the representation of peptide segments. Here, the widely used position-specific scoring matrix (PSSM) was employed to represent each peptide segment. The PSI-BLAST [42] with Swissprot [43] database was used to generate the PSSM profiles for each peptide segment. These profiles were further refined into a 540 (27 × 20)-dimension vector. The RAKEL algorithm was adopted to construct the model, where the base classification algorithm was RF or DT. The constructed model was also assessed by 5-fold cross-validation. The prediction results are listed in Table 5. The performance of such model was relatively low despite which base classification algorithm was selected. The aiming, coverage, accuracy and absolute true values were lower than 0.62, and the absolute false value was higher than 0.14. Such performance was greatly lower than that of RMTLysPTM. Moreover, the performance of the model on the four PTM types (Supplementary Table S1 and Supplementary Figure S2) was evidently lower than that of RMTLysPTM. Therefore, RMTLysPTM was much better than the model using PSSM features and RAKEL. The findings partly proved that the distribution features were more powerful than the PSSM features in recognizing the PTM types. Given the same prediction engine (RAKEL), the results in Table 5 showed that the models with distribution features were better than those with PSSM features, further confirming the results above.

### Performance of RMTLysPTM on protein Q16778

Protein Q16778 was a test protein in Qiu *et al*.’s study, and it was used as an independent testing dataset in some previous studies [23, 24, 26]. This protein was also employed to test the generalization ability of RMTLysPTM. The predicted and experimental results of the lysine sites in Q16778 are listed in Table 6. Sites 6–86 were correctly predicted. The aiming, coverage, accuracy, absolute true and absolute false values were 0.8250, 0.8250, 0.8167, 0.8000 and 0.0625, respectively. Although this performance was evidently lower than the training results (Table 1), it was still relatively high, suggesting that RMTLysPTM had a strong generalization ability.

**Table 6:** Comparison between predicted and experimental results on protein Q16778

Sites	Experimental result				Predicted result			
	Acetylation	Crotonylation	Methylation	Succinylation	Acetylation	Crotonylation	Methylation	Succinylation
6	√	√	x	x	√	√	x	x
12	√	√	x	x	√	√	x	x
13	√	√	x	x	√	√	x	x
16	√	√	x	x	√	√	x	x
17	√	√	x	x	√	√	x	x
21	√	√	x	x	√	√	x	x
24	√	√	x	x	√	√	x	x
25	x	x	x	x	x	x	x	x
28	x	x	x	x	x	x	x	x
29	x	x	x	x	x	x	x	x
31	x	x	x	x	x	x	x	x
35	x	√	x	x	x	√	x	x
44	x	x	x	x	x	x	x	x
47	x	x	√	x	x	x	√	x
58	x	x	√	x	x	x	√	x
86	√	x	√	x	√	x	√	x
109	x	x	√	x	x	x	x	x
117	x	x	x	x	√	x	x	x
121	√	√	x	x	√	x	x	√
126	x	x	x	x	x	x	x	√

### Comparison with previous models

This study adopted the lysine PTM sites in Qiu dataset. This dataset has become a benchmark dataset, which has been used to test the performance of all previous models, because it is the first dataset containing multiple types on lysine PTM sites. The previous models were tested by 5-fold cross-validation, and their overall performance is listed in Table 7. Among them, iMul-kSite provided the best 5-fold cross-validation results on Qiu dataset. Its accuracy and absolute true values reached 0.9270 and 0.8877, respectively. CNN + SGT ranked the second, mLysPTMpred and predML-Site provided nearly equal performance, whereas MLysPRED and iPTM-mLys had evidently lower performance than the above models. The performance of these models was compared with that of RMTLysPTM. For easy comparison, the overall performance of RMTLysPTM under two types of 5-fold cross-validation is listed in Table 7. Under the general 5-fold cross-validation, RMTLysPTM provided much higher performance than the existing models. For example, the absolute true value of RMTLysPTM was at least 10% higher than those of the other models. Furthermore, the aiming, coverage and accuracy values were evidently higher. Meanwhile, the absolute false value was remarkably lower than others at lower than 0.01, whereas those of the other models were higher than 0.02. The overall performance of RMTLysPTM under a strict 5-fold cross-validation was almost equal to that of iMul-kSite and higher than the performance of other previous models. These results indicated that RMTLysPTM was superior to all existing models. As mentioned in Introduction, iPTM-mLys, predML-Site, mLysPTMpred and iMul-kSite considered each type of lysine PTM sites individually and thus ignored the mutual influence of different types. In this case, they cannot use the information of one type to predict another one, which is the main reason why their performance was lower than that of RMTLysPTM. Although CNN + SGT and MLysPRED were constructed by directly using multi-label classification algorithms, the features of peptide segments were not very informative. The features used in these methods were mainly extracted from a single sequence and did not include the label information. RMTLysPTM adopted the features by deeply analyzing the distribution of 2-residue in the training dataset on four labels, which were more informative than those in CNN + SGT and MLysPRED. Therefore, RMTLysPTM had higher performance than CNN + SGT and MLysPRED.

**Table 7:** Five-fold cross-validation results of different models on Qiu dataset

Model	Aiming	Coverage	Accuracy	Absolute true	Absolute false
iPTM-mLys [3]	0.6978	0.7454	0.6837	0.6092	0.1340
mLysPTMpred [24]	0.8482	0.8656	0.8373	0.7973	0.0666
CNN + SGT [26]	0.8391	0.8391	0.8275	0.8521	0.0427
predML-Site [23]	0.8534	0.8658	0.8418	0.8056	0.0641
iMul-kSite [25]	0.9318	0.9613	0.9270	0.8877	0.0297
MLysPRED [27]	0.8082	0.8519	0.7876	0.7002	0.1078
RMTLysPTM^a^	0.9943	0.9949	0.9934	0.9909	0.0023
	0.9306	0.9604	0.9291	0.8955	0.0275

^a^RMTLysPTM is tested by two types of 5-fold cross-validation.

The performance of several previous models on protein Q16778 was tested, as in Performance of RMTLysPTM on protein Q16778 section. The overall performance is listed in Table 8. The test results of RMTLysPTM were included in this table for easy comparison. predML-Site provided the highest performance with the best values on all five measurements. The performance of RMTLysPTM was slightly lower than those of predML-Site and mLysPTMpred. However, it was still competitive. RMTLysPTM only correctly predicted one lysine site less than predML-Site because 20 lysine sites were only present in protein Q16778. Therefore, few lysine sites increased the prediction contingency.

**Table 8:** Performance of different models on protein Q16778

Model	Aiming	Coverage	Accuracy	Absolute true	Absolute false
iPTM-mLys [3]	0.6750	0.6500	0.6250	0.5500	0.1500
mLysPTMpred [24]	0.8833	0.8750	0.8583	0.8000	0.0600
CNN + SGT [26]	0.6500	0.6500	0.6500	0.8500	0.0500
predML-Site [23]	0.9000	0.8750	0.8750	0.8500	0.0500
RMTLysPTM	0.8250	0.8250	0.8167	0.8000	0.0625

### Test results on CPLM training and testing datasets

In [27], new lysine PTM data derived from CPLM 4.0 were constructed. They contained two datasets, which were called CPLM training and testing datasets. Here, RMTLysPTM was evaluated on these two datasets. The sliding window size was set to 24 to give a fair comparison, as used in [27]. The 5-fold cross-validation results on the CPLM training dataset are listed in Table 9. The aiming, coverage, accuracy, absolute true and absolute false values were 0.9983, 0.9986, 0.9977, 0.9958 and 0.0011, respectively. Such performance was relatively similar to that on Qiu dataset. Furthermore, the RMTLysPTM built on the CPLM training dataset was applied to the CPLM testing dataset, and its performance is listed in Table 10. The overall measurements were 0.9440, 0.9269, 0.9144, 0.8688 and 0.0431, respectively. Such performance was lower than that on the CPLM training dataset. However, the declined degree was not very large. The performance on the CPLM testing dataset was still relatively high, suggesting the strong generalization ability of RMTLysPTM. Given that the CPLM testing dataset was much larger than the independent testing set in Performance of RMTLysPTM on protein Q16778 section, such test results were more reliable.

**Table 9:** Performance of two models on the CPLM training dataset

Model	Aiming	Coverage	Accuracy	Absolute true	Absolute false
RMTLysPTM	0.9983	0.9986	0.9977	0.9958	0.0011
MLysPRED [27]	0.7998	0.8308	0.7667	0.6723	0.1397

The performance of RMTLysPTM on CPLM training and testing datasets was compared with those of the other models to show its superiority. The 5-fold cross-validation results of MLysPRED on the CPLM training dataset are provided in Table 9. Its performance was evidently lower than that of RMTLysPTM. The gaps on accuracy and absolute true values were more than 20%. Its performance on the CPLM testing dataset is provided in Table 10. On the testing dataset, the gap between RMTLysPTM and MLysPRED was not very large. The accuracy was only about 2% lower, and the absolute true was about 5% lower. Table 10 shows the performance of the other two methods (iMul-kSite and predML-Site) on the CPLM testing dataset, which was directly obtained from [27]. Their performance was much lower than those of RMTLysPTM and MLysPRED. These comparison results suggested that RMTLysPTM was superior to the above previous models.

**Table 10:** Performance of different models on the CPLM testing dataset

Model	Aiming	Coverage	Accuracy	Absolute true	Absolute false
RMTLysPTM	0.9440	0.9269	0.9144	0.8688	0.0431
MLysPRED [27]	0.9221	0.9498	0.8963	0.8146	0.0682
iMul-kSite [25]	0.4881	0.4548	0.4402	0.3859	0.1992
predML-Site [23]	0.5503	0.5132	0.4967	0.4371	0.1952

### Multi-label versus binary

For the lysine PTM prediction problem, most models were designed as binary classifiers. In fact, the previous models, such as iPTM-mLys, predML-Site, mLysPTMpred and iMul-kSite, were designed in this manner. The individual binary classifiers for four types of lysine PTM sites were combined as the final multi-label classifiers. Such scheme had an evident defect, that is, the mutual influence of different types of lysine PTM sites was neglected. In general, a good multi-label classifier should provide better performance on each type of lysine PTM sites than the binary classifier on the same PTM type. Some tests were conducted to confirm this hypothesis.

The binary classifier was built using the same peptide segment representation in Feature construction section. The following classic binary classification algorithms were adopted as the prediction engine: support machine vector [44], RF [39] and Bayesian network. The 5-fold cross-validation results of these binary classifiers are listed in Table 11. At a glance, the classifiers provided good performance for each type of lysine PTM site. However, such performance was much lower than that of RMTLysPTM on each PTM type (Table 2 and Figure 5). This result implied that RMTLysPTM is a qualified multi-label classifier, and overall consideration of four types of lysine PTM sites can improve the prediction accuracy for each PTM type.

**Table 11:** Performance of binary classifiers on four types of lysine PTM sites in Qiu dataset

Classification algorithm	Lysine PTM type	Accuracy	Precision	Sensitivity	Specificity	F1-score	AUROC	AUPR
Support vector machine	Acetylation	0.8598	0.8809	0.8978	0.7970	0.8886	0.8474	0.8623
	Crotonylation	0.9961	0.9049	0.8771	0.9982	0.8880	0.9377	0.7943
	Methylation	0.9955	0.9752	0.7891	0.9995	0.8348	0.8943	0.7704
	Succinylation	0.9269	0.8116	0.7218	0.9739	0.7584	0.8478	0.7019
Random forest	Acetylation	0.8451	0.8367	0.9412	0.6872	0.8851	0.9028	0.9297
	Crotonylation	0.9975	0.9521	0.9014	0.9992	0.9209	0.9851	0.9630
	Methylation	0.9965	0.9905	0.8323	0.9998	0.8645	0.9390	0.8703
	Succinylation	0.8957	0.9225	0.7757	0.9976	0.8104	0.9132	0.8253
Bayesian network	Acetylation	0.8193	0.8857	0.8272	0.8055	0.8446	0.9052	0.9303
	Crotonylation	0.9968	0.8762	0.9545	0.9976	0.9134	0.9765	0.8817
	Methylation	0.9969	0.9905	0.8480	0.9998	0.8725	0.9239	0.8415
	Succinylation	0.9374	0.8051	0.7689	0.9764	0.7737	0.9063	0.8287

### Web server and user guide

**Figure 7:**
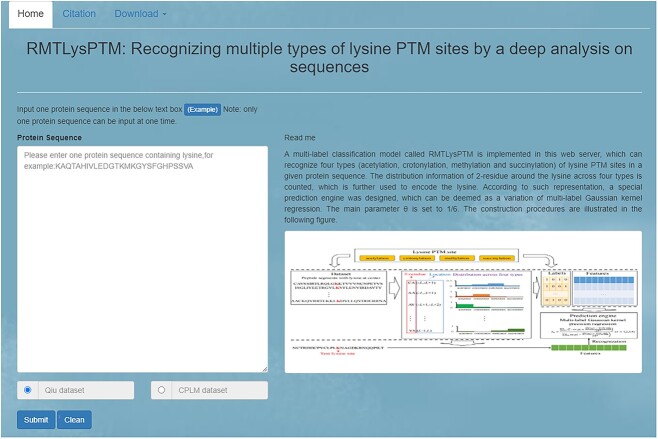
Home page of the web server.

For easy use of RMTLysPTM, a user-friendly web server with the same name was set up, which can be accessed at http://119.3.127.138/. The home page is shown in Figure 7. Users can submit a protein sequence and the web server can give the recognition result for each lysine site in this sequence. A step-by-step guide was provided below.

Step 1. Open the web server at http://119.3.127.138/ and input the protein sequence in the text box. Examples can be obtained by clicking the ‘Example’ button above the text box.

Step 2. Select the prediction model based on Qiu dataset or CPLM dataset below the text box.

Step 3. After inputting the protein sequence, click ‘Submit’ button to upload the sequence. The recognition result will be displayed in a new web page. Users can click ‘Clean’ to clear the current input and give a new input.

Step 4. In the result page, the locations of lysine sites in the sequence and the identified PTM types are listed. Using the ‘Back’ button, users can return the home page.

In this web server, the reference can be found by clicking ‘Citation’ button at the top of home page. The underlying dataset and codes are also provided. Users can click ‘Download’ button to obtain them. A notable detail that the feature representations of peptide segments were used to train the final classification model rather than those used in 5-fold cross-validation.

### Limitations and future work

This model has some limitations. As shown in Figures 2 and 3, the lysine sites modified by four types were not equal. The sites modified by acetylation was evidently much more than those modified by the other three types. In another word, the Qiu and CPLM datasets were imbalanced. This problem was not considered when constructing RMTLysPTM. The addition of oversampling or undersampling techniques may improve the generalization ability of the model. Meanwhile, the features to represent peptide segment lacked diversity. When constructing RMTLysPTM, the distribution of 2-residue on the four types of lysine PTM sites was overemphasized, and the essential properties of the peptide segment may have been ignored. The model could be further improved by combining them. Finally, many lysine PTM types have been detected. For example, CPLM 4.0 collected nearly 30 types of lysine PTM sites. This study followed the previous ones and only investigated four types. If more lysine PTM types were employed, the labels could have been increased. Dealing with many labels is a challenging problem. In the future, this work will be continued to overcome the above limitations.

## CONCLUSION

This study presented a multi-label classification model called RMTLysPTM to recognize the types of lysine PTM sites. Deep analysis was conducted to extract the distribution information of 2-residue with fixed location across four types to access the informative features of peptide segments containing lysine at the center. Powerful features were constructed on the basis of such information, and an efficient prediction engine that can capture the traits of the constructed features was designed. Such model is very powerful, and it can be an efficient tool to recognize types of lysine PTM sites. A user-friendly web server (http://119.3.127.138/) was set up for easy usage, along with the codes and underlying datasets.

**Key Points**

- This study proposed a new multi-label classification model, RMTLysPTM, for recognizing multiple types of lysine PTM sites.
- Such model counted the distribution information of 2-residue at fixed location across four types of lysine PTM sites, which was used to encode each peptide segment with lysine at center.
- A new prediction engine was designed, which can capture the traits of the representations of peptide segments.
- Test results on two different datasets have shown high performance of RMTLysPTM.
- RMTLysPTM was superior to all previous models and was also better than other models that incorporated widely used methods and features.

## Supplementary Material

Figure_S1_bbad450

Figure_S2_bbad450

Table_S1_bbad450

## Data Availability

The Qiu dataset is available at http://www.jci-bioinfo.cn/iPTM-mLys or in the paper of Qiu et al. at https://doi.org/10.1093/bioinformatics/btw380. The CPLM training and testing datasets are available at http://47.100.136.41:8181/dataSet. The web server for RMTLysPTM can be accessed at http://119.3.127.138/.

## Author Biographies

**Lei Chen** is an associate professor at the College of Information Engineering of Shanghai Maritime University. His interests include bioinformatics, computational biology and machine learning.

**Yuwei Chen** is a PhD candidate at the Shanghai maritime University. Her interests include machine learning and bioinformatics.
