# Supplementary figures and images for: RMTLysPTM: recognizing multiple types of lysine PTM sites by deep analysis on sequences
Source: Brief Bioinform. 2023 Dec 8;25(1):bbad450. doi: 10.1093/bib/bbad450 (PMC10783864; doi:10.1093/bib/bbad450)

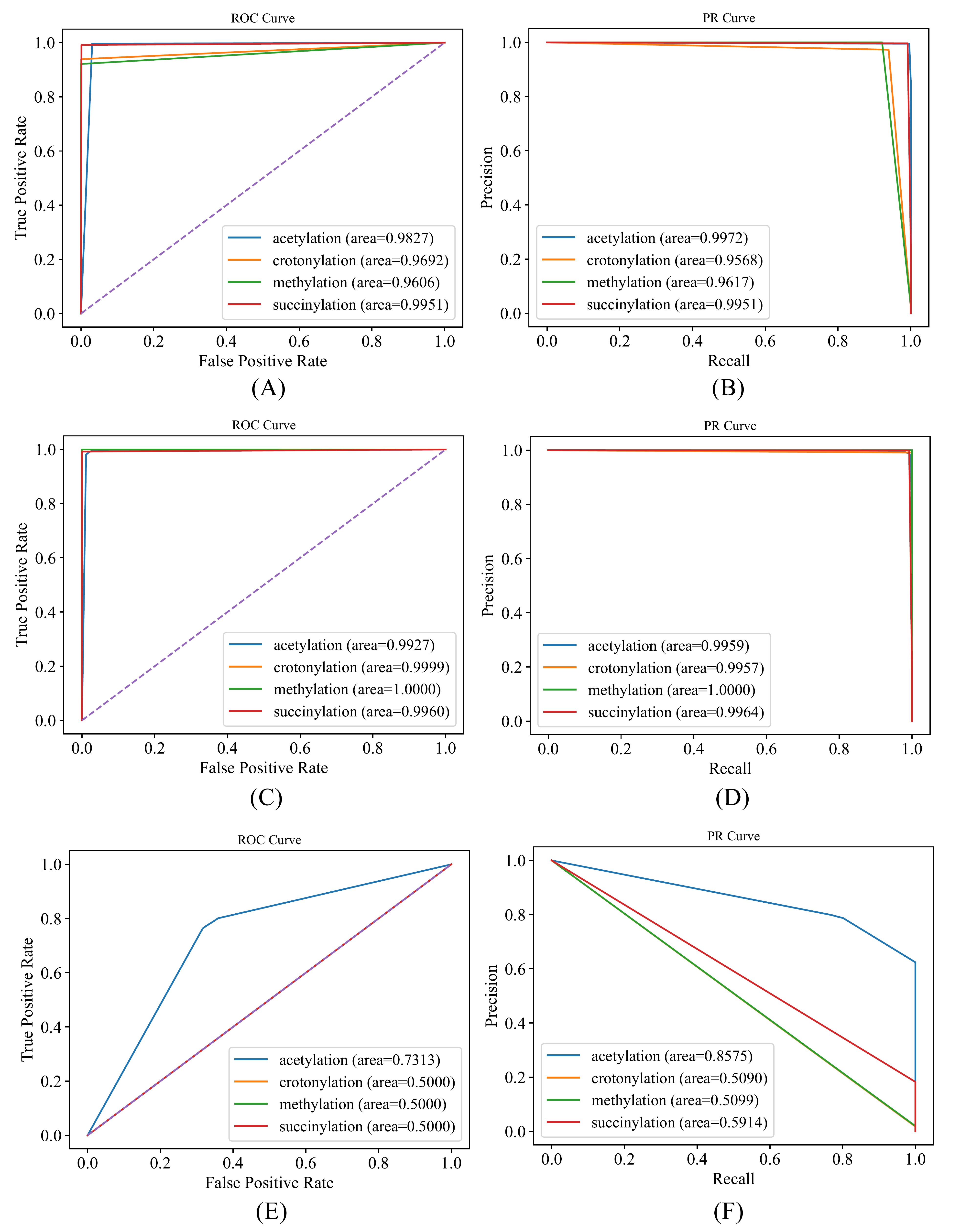

Supplement: Figure_S1_bbad450 [file figure_s1_bbad450.jpeg]

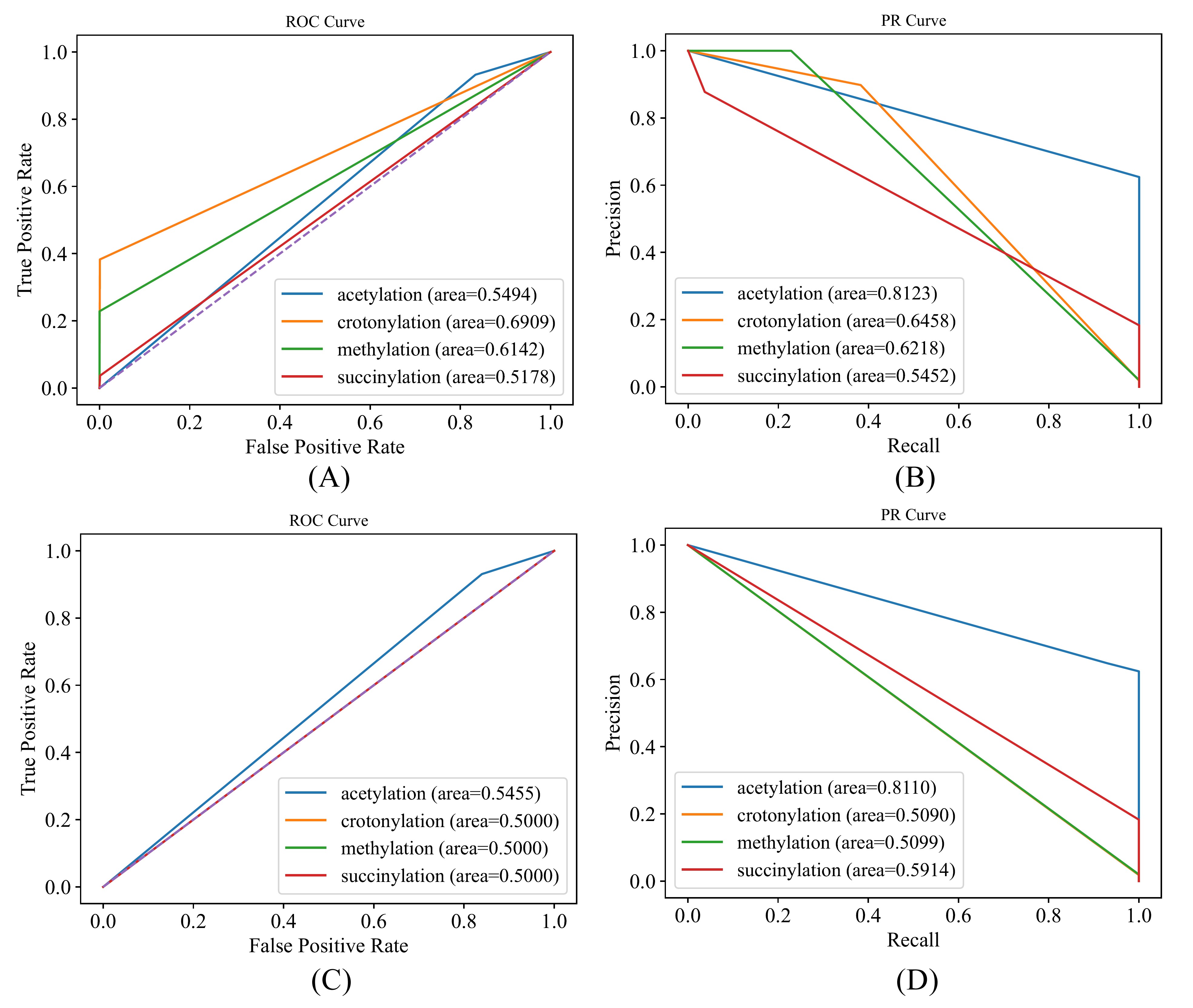

Supplement: Figure_S2_bbad450 [file figure_s2_bbad450.jpeg]
