# Supplementary material for: RMTLysPTM: recognizing multiple types of lysine PTM sites by deep analysis on sequences
Source: Brief Bioinform. 2023 Dec 8;25(1):bbad450. doi: 10.1093/bib/bbad450 (PMC10783864; doi:10.1093/bib/bbad450)
Supplement: Table_S1_bbad450 [file table_s1_bbad450.docx]

**Table S1.** Performance of various models on four types of lysine PTM sites in Qiu dataset.

| **Feature** | **Prediction engine** | **Type** | **Accuracy** | **Precision** | **Sensitivity** | **Specificity** | **F1-score** |
| --- | --- | --- | --- | --- | --- | --- | --- |
| Distribution feature | ML-GKR | acetylation | 0.9922 | 0.9950 | 0.9960 | 0.9692 | 0.9955 |
|  |  | crotonylation | 0.9978 | 0.9730 | 0.9391 | 0.9993 | 0.9558 |
|  |  | methylation | 0.9978 | 1.0000 | 0.9213 | 1.0000 | 0.9590 |
|  |  | succinylation | 0.9970 | 0.9966 | 0.9914 | 0.9973 | 0.9940 |
|  | RAKEL  (Random forest) | acetylation | 0.9797 | 0.9710 | 0.9972 | 0.9500 | 0.9839 |
|  |  | crotonylation | 0.9997 | 0.9829 | 1.0000 | 0.9997 | 0.9914 |
|  |  | methylation | 1.0000 | 1.0000 | 1.0000 | 0.9998 | 1.0000 |
|  |  | succinylation | 0.9983 | 0.9983 | 0.9923 | 0.9969 | 0.9953 |
|  | RAKEL  (Decision tree) | acetylation | 0.7410 | 0.7876 | 0.8011 | 0.6408 | 0.7943 |
|  |  | crotonylation | 0.9820 | 0.0000 | 0.0000 | 1.0000 | 0.0000 |
|  |  | methylation | 0.9801 | 0.0000 | 0.0000 | 1.0000 | 0.0000 |
|  |  | succinylation | 0.8172 | 0.0000 | 0.0000 | 1.0000 | 0.0000 |
| PSSM feature | RAKEL  (Random forest) | acetylation | 0.6445 | 0.6501 | 0.9323 | 0.1665 | 0.7660 |
|  |  | crotonylation | 0.9881 | 0.898 | 0.3826 | 0.9992 | 0.5366 |
|  |  | methylation | 0.9847 | 1.0000 | 0.2283 | 1.0000 | 0.3718 |
|  |  | succinylation | 0.8230 | 0.8776 | 0.0368 | 0.9988 | 0.0706 |
|  | RAKEL  (Decision tree) | acetylation | 0.6412 | 0.6480 | 0.9308 | 0.1603 | 0.7641 |
|  |  | crotonylation | 0.982 | 0.0000 | 0.0000 | 1.0000 | 0.0000 |
|  |  | methylation | 0.9801 | 0.0000 | 0.0000 | 1.0000 | 0.0000 |
|  |  | succinylation | 0.8172 | 0.0000 | 0.0000 | 1.0000 | 0.0000 |
